# Artificial Neural Networks Model for Predicting Type 2 Diabetes Mellitus Based on *VDR* Gene *FokI* Polymorphism, Lipid Profile and Demographic Data

**DOI:** 10.3390/biology9080222

**Published:** 2020-08-13

**Authors:** Ma’mon M. Hatmal, Salim M. Abderrahman, Wajeha Nimer, Zaynab Al-Eisawi, Hamzeh J. Al-Ameer, Mohammad A. I. Al-Hatamleh, Rohimah Mohamud, Walhan Alshaer

**Affiliations:** 1 Department of Medical Laboratory Sciences, Faculty of Applied Health Sciences, The Hashemite University, Zarqa 13133, Jordan; Zaynab@hu.edu.jo; 2 Department of Biology, Faculty of Science, The Hashemite University, Zarqa 13133, Jordan; salim_dr_1954@hotmail.com (S.M.A.); as1989_jo@yahoo.com (W.N.); 3 Department of Biology and Biotechnology, American University of Madaba, Madaba 11821, Jordan; h.alameer@aum.edu.jo; 4 Department of Immunology, School of Medical Sciences, Universiti Sains Malaysia, Kubang Kerian, Kelantan 16150, Malaysia; alhatamleh@student.usm.my (M.A.I.A.-H.); rohimahm@usm.my (R.M.); 5 Hospital Universiti Sains Malaysia, Kubang Kerian, Kelantan 16150, Malaysia; 6 Cell Therapy Center (CTC), The University of Jordan, Amman 11942, Jordan

**Keywords:** T2DM, diabetes, diabetic Jordanians, *FokI* polymorphism, *VDR* gene, FNN, neural network, lipids, medical databases

## Abstract

Type 2 diabetes mellitus (T2DM) is a multifactorial disease associated with many genetic polymorphisms; among them is the *FokI* polymorphism in the vitamin D receptor (*VDR*) gene. In this case-control study, samples from 82 T2DM patients and 82 healthy controls were examined to investigate the association of the *FokI* polymorphism and lipid profile with T2DM in the Jordanian population. DNA was extracted from blood and genotyped for the *FokI* polymorphism by polymerase chain reaction (PCR) and DNA sequencing. Lipid profile and fasting blood sugar were also measured. There were significant differences in high-density lipoprotein (HDL) cholesterol and triglyceride levels between T2DM and control samples. Frequencies of the *FokI* polymorphism (CC, CT and TT) were determined in T2DM and control samples and were not significantly different. Furthermore, there was no significant association between the *FokI* polymorphism and T2DM or lipid profile. A feed-forward neural network (FNN) was used as a computational platform to predict the persons with diabetes based on the *FokI* polymorphism, lipid profile, gender and age. The accuracy of prediction reached 88% when all parameters were included, 81% when the *FokI* polymorphism was excluded, and 72% when lipids were only included. This is the first study investigating the association of the *VDR* gene *FokI* polymorphism with T2DM in the Jordanian population, and it showed negative association. Diabetes was predicted with high accuracy based on medical data using an FNN. This highlights the great value of incorporating neural network tools into large medical databases and the ability to predict patient susceptibility to diabetes.

## 1. Introduction

Diabetes mellitus (DM) is a metabolic disease characterized by chronic hyperglycemia accompanied by the failure in the metabolism of lipids, carbohydrates and proteins. Type 2 DM (T2DM) is associated with a group of plasma lipid and lipoprotein abnormalities, including reduced high-density lipoprotein (HDL) cholesterol, a predominance of small dense low-density lipoprotein (LDL) cholesterol particles and high triglycerides (TG) levels. These abnormalities occur in many patients despite normal LDL cholesterol levels. These changes are also a major feature of the insulin resistance syndrome, which underlies many cases of T2DM. In fact, individuals with diabetes often exhibit different lipid risk factors that include higher levels of total cholesterol (TC), LDL and TG, as well as lower levels of HDL [1].

On the other hand, the role of vitamin D in the pathogenesis and prevention of T2DM has gained widespread interest [2]. Vitamin D has major roles in the regulation of insulin secretion from β-cells and the homeostasis of calcium [3] and regulation of growth and differentiation of diverse types of cells [4]. These roles are partially mediated by binding of vitamin D to its specific receptors. The resulted complex then acts as an active transcription factor that controls the expression of about 200 genes including the insulin gene. Hence, any variations in the *VDR* gene could affect the insulin level and might lead to diabetes [3]. The vitamin D receptor (*VDR*) gene is located on chromosome 12 (12q14). This gene includes six untranslated exons (exons 1a–1f) (alternatively spliced) and eight protein-coding exons (exons 2–9) [5].

In recent years, several single nucleotide polymorphisms (SNPs) have been described in the *VDR* gene that can alter *VDR* protein activity, including *BsmI* (A > G, rs1544410), *ApaI* (A > C, rs7975232), *TaqI* (T > C, rs731236) and *FokI* (C > T, rs2228570). The *FokI* polymorphism (ATG→ACG) in exon 2 of the *VDR* gene is considered as the only known locus affecting the structure of the *VDR* protein [6]. This transition from T to C allele would eliminate the first potential ATG translation start site and allow a second one, 9 bp downstream, to be used [2]. The T allele encodes a protein that contains 427 amino acids while the C allele encodes a protein containing 424 amino acids. The shorter *VDR* protein variant seems to function more effectively in binding with the active form of vitamin D (1,25-dihydroxyvitamin D). The increase in the level of vitamin D reduces the risk of T2DM by enhancing pancreatic β-cell secretion function, ultimately improving insulin resistance. This biological mechanism could explain the correlation between the T allele of the *FokI* polymorphism and susceptibility to T2DM [6].

Many studies have reported the association between the *FokI* polymorphism and T2DM [2,4,7,8,9,10,11], while few studies have reported the lack of association [6,12,13]. To date, there are no data on the association of the *FokI* polymorphism with T2DM in the Jordanian population, although the prevalence of diabetes in Jordan is among the highest in the world, which makes it a big public health burden. The prevalence of diabetes elevated from 6.3% in 2002 to 7.4% in 2004 in Jordan’s population [12]. A study published in 2008 by Ajlouni [13] showed a 31.5% increase in the prevalence of diabetes among Jordanians with an age of 25 years or older in comparison with a similar survey conducted in 1994. It is expected that, at the end of 2050, about 1 to 3 million people in Jordan will have diabetes, hypertension or increased blood cholesterol depending on the changes in disease prevalence and the growth of the population [14]. According to the latest data by the International Federation of Diabetes, the prevalence of diabetics among Jordanian adults is 9.9% with 544,200 cases [15].

Although insulin resistance (IR) is known as a powerful predictor of T2DM development and a therapeutic target once hyperglycemia is present, distribution curves of insulin sensitivity as measured by the euglycemic-hyperinsulinemic clamp show that people with T2DM sit within the range of the non-diabetic distribution, but toward the lower range [16], which needs a health specialist for such examination. Since IR calculation is dependent on high levels of blood sugar, TG and LDL, as well as low HDL level, using lipid profile can be considered the simplest and most acceptable because TG and HDL were shown to be correlated with IR [17].

DM is one of the biggest diseases in all age groups. The earlier diagnosis we get, the better we can handle it. Machine learning, according to their regular physical examination results, can help people make a preliminary decision about DM and it can serve as a guide for doctors [18,19,20]. Applying machine learning and data mining methods in DM research is a crucial approach for using vast amounts of available diabetes-related data for information extraction [21,22,23]. The severe social impact of the specific disease makes DM one of the top priorities of medical science research, which eventually generates vast amounts of data. Therefore, machine learning and data mining methods in DM are certainly of great concern when it comes to diagnosis, management and other related aspects of clinical administration [18,24,25]. The most critical problems for machine learning systems are how to pick the appropriate features and the right classifier. Numerous algorithms have recently been used to predict diabetes including the standard approaches of machine learning (i.e., feed-forward neural network (FNN)) [18,19,20,26,27].

Therefore, our objectives in this study were to investigate whether the *FokI* polymorphism in the *VDR* gene is associated with T2DM susceptibility, evaluate the frequency and prevalence of this reported SNP in T2DM among Jordanians and to evaluate the relationship between this polymorphism and their lipid profile. Moreover, a feed-forward neural network (FNN) was used to predict the persons with diabetes based on the *FokI* polymorphism, lipid profile, gender and age as inputs.

## 2. Materials and Methods

### 2.1. Study Design and Population

Adult Jordanians with T2DM and healthy controls were invited to participate in a case-control study from 1 January 2016 to 1 December 2018 at The Hashemite University, Zarqa, Jordan. All patients with T2DM were chosen based on medical examination history, while healthy controls were with no clinical history of T2DM. Patients with T2DM were diagnosed according to the World Health Organization (WHO) criteria [28] by specialized pathologists and had received medical care for couple of years.

There were several inclusion and exclusion criteria used in this study. Inclusion criteria were healthy individuals, diabetic patients with or without complications, able to give consent and above 18 years of age. While exclusion criteria were pregnant female, not be able to consent and less than 18 years old. Participants were assessed for previously diagnosed diabetes by asking if have ever been told that they have diabetes by a doctor or health care professional. Those who answered by yes, excluding gestational DM, were defined as having previously diagnosed diabetes. A subject was deemed affected by DM if this diagnosis was known to the patient or if his or her condition complies with the WHO definition; fasting serum glucose was 7 mmol/L (126 mg/dL) or more. Thus, diabetes was defined as hyperglycemia, requiring antidiabetic drugs or testing blood glucose level ≥7.0 mmol/L [13].

This study was approved by the Institutional Review Board Committee (IRB) at The Hashemite University (reference number: 9/1/2017/2018). Informed consents were obtained from all the participants. Data including age and family history of T2DM were obtained from all participants.

### 2.2. Blood Collection and Chemistry Tests

Blood samples were collected from T2DM patients and healthy controls in plain and EDTA tubes after fasting overnight (8–10 h). Palin tubes were centrifuged, and serum was used to analyze fasting blood sugar (FBS) and lipid profile parameters, including TC, TG and HDL by using Cobas C111 analyzer (Roche Diagnostics, Indianapolis, IN, USA). The LDL was calculated according to the equation of Friedewald [29]:

LDL=TC−HDL−(TG/5)

### 2.3. DNA Extraction and Quantification

Genomic DNA was extracted from EDTA blood using the Wizard genomic DNA purification kit (Promega Corporation, Madison, WI, USA) according to the manufacturer’s instructions. The quantity and the quality of the extracted DNA were assessed by spectrophotometric quantification using a Nano-Drop™ 2000/2000c Spectrophotometer (Thermo Fisher Scientific, Waltham, MA, USA). DNA absorbs UV light at a 260 nm, and DNA concentration (ng/mL) is estimated in each DNA sample depending on the amount of UV light absorbed by the sample. To confirm the purity of the DNA sample, the ratio of OD260/OD280 was calculated. A ratio between 1.8 and 2.0 values indicates that the UV light was absorbed by nucleic acids. After quantification, all the samples were stored at −20 °C for further analysis.

### 2.4. Polymerase Chain Reaction (PCR)

In this study, exon 2 was amplified to study the *FokI* polymorphism in the *VDR* gene. The genomic DNA was amplified using a pair of primers (forward primer 5′-AGCTGGCCCTGGCACTGACTCTGCTCT-3′ and reverse primer 5′-ATGGAAACACCTTGCTTCTTCTCCCTC-3′). According to the certificate of analysis provided by Gene Link (Hawthorne, NY, USA), each vial of forward and reverse primer contained 24.6 nmole. The stock concentration of primers (100 μM) was prepared, which was then used to prepare 10 μL of working solution. The PCR reaction of exon 2 was made in 0.2 mL PCR tubes with a final mix volume of 30 μL. The 30 μL of PCR mixture was composed of 6.0 μL master mix (DNA polymerase, proofreading enzyme, buffer, MgCl_2_, dNTPs, yellow and blue dyes, bovine serum albumin and density reagent), 1.0 μL of each primer, 1.0 μL of template DNA and 21 μL of nuclease-free water. The PCR program was as follows: initial denaturation at 95 °C for 3 min, then 45 cycles of 30 s at 95 °C (denaturation), 40 s at 53.3 °C (annealing) and 50 s at 72 °C (extension), followed by a final extension at 72 °C for 10 min. The resultant amplified sequence length of the *FokI* polymorphism was 265 bp [6].

### 2.5. Agarose Gel Electrophoresis

To evaluate PCR amplification, the electrophoresis technique was used to verify the migration of DNA fragments in an agarose gel. For PCR electrophoresis, an agarose gel was made with a concentration of 2.5% (*m*/*v*). It was prepared by boiling 2.5 g agarose with 100 mL 1X tis-borate-EDTA (TBE) buffer and stained with 5.0 μL red safe stain. Then, 3 μL of PCR products were loaded and migration of fragments in the agarose gel was performed under voltage conditions of 120 V for 30 min. To compare the molecular weight of DNA fragments, a 100 bp DNA ladder was used. For agarose gel visualization, a UV Transilluminator was used (UVP Bioimaging System, Upland, CA, USA).

### 2.6. DNA Sequencing

DNA sequencing of selected samples was used to confirm the genotypes of the *FokI* polymorphism. Dideoxynucleotides (ddNTPs) are readily incorporated into a growing DNA chain, but lacking a 3′ hydroxyl group that leads the chain to continue and terminate the polymerization effectively [30].

After amplification of the *VDR* gene fragments through PCR, the resultant products had to be purified to eliminate and neutralize PCR residuals. The PCR purification step was done by using the ExoSAP-IT™ kit (Applied Biosystems, Waltham, MA, USA) according to the manufacturer’s instructions. In this reaction, 1.0 μL of ExoSAP reagent was added to 2.5 μL of the PCR product. Once the reaction came out of thermocycler, the product was diluted before sequencing by adding 20 μL of water.

After purification of the PCR products, DNA sequencing was done at Macrogen Inc. (Seoul, South Korea) by using the ABI 3730*xl* DNA Analyzer (Applied Biosystems, Waltham, MA, USA) with big dye terminator version 3.1 kit. The determined sequences that were aligned with the reference sequence of the *VDR* gene were downloaded from the NCBI-nucleotide database (accession number: NG_008731) [31].

### 2.7. Statistical Analysis

The statistical analysis was performed using SPSS version 16.0 (SPSS Inc., Chicago, IL, USA). Genotype distributions between groups were calculated and Hardy–Weinberg equilibrium (HWE) was performed in order to verify if the control group of the present study was under the assumptions of this law. Therefore, direct counting of the observed genotype frequencies in this group was made, and then the expected values were calculated. For example, if the alleles C and T are present in a genotype, where the allele frequency for C allele is “p” and for T allele is “q” (q=1−p), the expected values for the CC, CT and TT genotypes will be p2, 2pq and q2 [16]. The odds ratio (OR) at a 95% confidence interval (CI) was determined by logistic regression. Comparisons of biochemical data between groups were made using 2-tailed *t*-test. The relationship between investigated polymorphism genotypes and lipid profile parameters was made using the R×C chi-square test. The level of statistical significance was set at *p*-value ≤0.05.

### 2.8. Multilayer FNN

For the aim of predicting diabetes, a multilayer FNN was built using Python, Keras Toolbox™, R2013b [32,33]. Data were divided for a training set (80%) and testing set (20%). A multilayer FNN was chosen for its comprehensive foundation and because it is one of the widely used models in many practical applications degrading elegantly in the presence of increasing amounts of noise [34,35,36].

The input–output training set of the multilayer FNN contained the *FokI* polymorphism, lipid profile, gender and age as inputs, and the diabetes status (1 for person with diabetes and 0 for person without diabetes) as output. Label-encoders and hot-encoders were used for the *FokI* polymorphism and gender, and thus the number of input nodes were converted to ten (three for the *FokI* polymorphism, two for gender and one node for each other input). Moreover, the best conditions of optimizer type and the number of epochs for each computation process were determined using grid search.

Multilayer FNN consists of an input layer of nodes, an output layer of nodes and one or more hidden layers (2 hidden layers in our case). The hidden layers are placed between the input and the output layer. Each layer consists of one or more processing nodes (10 nodes for the input layer, 6 for the first hidden layer, 6 for the second hidden layer and 1 for the output layer). The output of the node from one layer is fully connected (dense layers) to one or more nodes of the next layer. Each node implements a weighted (w) sum of its inputs (U) and a bias (b), which is then non-linearly transferred to one or more nodes of the next layer. Here, U and w are vectors that contain multiple components. Thus for the given example, a weighted and biased input (U∗w1+b1) is non-linearly transferred with a rectified linear unit (ReLU) activation function (H=ReLU(U∗w1+b1)), by the hidden layer as an input for the next layer which again is weighted, and biased and non-linearly transferred to the output (Y) by a sigmoid function (Y=sig (H∗w2+b2)) [32,33,37].

Before using the network for classification purposes, the multilayer FNN needs to be trained. The training is achieved by feeding the network with the training set. The aim of the training is to estimate the weights and bias values at every node of the network such that the trained network satisfactorily relates every input-output data from the training set. Such a trained multilayer FNN is capable to compute a unique output for wide range of inputs [32,33,37].

Thus effectively, U and Y, both vectors, were used to train the multilayer FNN model in predicting the diabetes status from other different inputs. The model with an input, an output and two hidden layers was trained with sufficient amount of input-output datasets (~132 samples) will instantly predict the diabetes status for an arbitrary set of input values measured in time [32,33,37,38,39]. The model was then used to predict the output of the testing list.

## 3. Results

### 3.1. Demographic and Clinical Data

A total of 164 random unrelated Jordanian individuals were involved in this study; 82 patients with T2DM and 82 healthy controls. The patient and control groups were matched for both age and gender. The average (standard deviation (SD)) of age for T2DM patients and healthy controls were 53.6 (9.8) years and 49.5 (12.8) years, respectively. Based on a *t*-test, there is no significant difference between the two groups. There were significant differences in HDL and TG levels between T2DM and control samples (Table 1). Baseline data of the study subjects is available in the Supplementary Materials (Table S1).

### 3.2. Genotyping of the FokI Polymorphism

**Figure 1:**
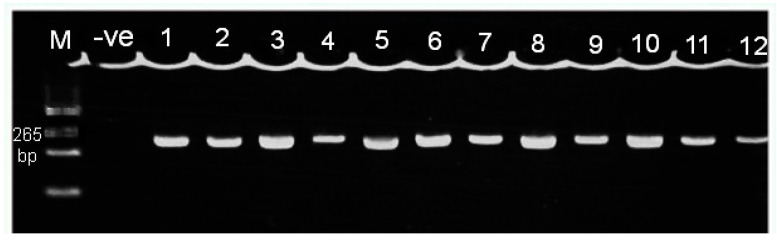
Electrophoresis of PCR product of *VDR* exon 2 region which contains the *FokI* polymorphism on 2.5% agarose gel. Lane M: DNA marker (100 bp); lane-ve: negative control: lane 1–12, samples with 265 bp bands.

After PCR, a 265 bp fragment of genomic DNA was observed via electrophoresis (Figure 1). Then, the genotyping of the *FokI* polymorphism was conducted using DNA sequencing technique. The frequencies of different genotypes are shown in Table 2.

Expected and observed HWE values for control samples are shown in Table 3. In order to check HWE, a comparison between the observed and expected genotype frequencies in the *FokI* polymorphism was carried out based on the χ^2^ test. The observed genotypes frequencies did not deviate significantly from those expected under the assumptions of HWE (Table 3).

### 3.3. Comparison of the Genotypes and Alleles

There were no significant differences (*p* > 0.05) upon comparison of the genotypes and alleles of the *FokI* polymorphism between T2DM patients and the control group (Table 4). Table S2 in the Supplementary Materials summarizes the logistic regression model and using it to calculate OR at 95% CI to describe the strength of association. In logistic regression the OR represents the constant effect of a predictor X on the likelihood that one outcome will occur. No significant difference in genotype distribution was observed between patients and controls (Table S2). Furthermore, no significant association was noted between T2DM patients and the controls when the samples were sub-grouped by gender (Table S3). The web tool “snpStats” was used in analysis of these associations.

### 3.4. Comparative Analyses of the Lipid Profile

The *t*-test was used to determine whether there is a significant difference in clinical parameters between the means of the two groups. Table 5 shows significant differences in the means of all lipid parameters between T2DM patients and control samples (*p* < 0.05). The Kolmogorov–Smirnov test was performed to assess the normality of data. Based on the *p*-value, data for all groups does not differ significantly from that which is normally distributed.

The main question in this study was “is there any association between hyperlipidemia (in lipid profile) and *FokI* polymorphism as categories?” If the question was to evaluate the differences between lipids levels among different genotypes, then ANOVA is an appropriate statistical test. However, in this case it just identifies if the lipids levels are different or not, and it will not show if there is an association between hyperlipidemia and different genotypes. Nevertheless, the ANOVA analysis was performed for different lipid parameters among different genotypes for T2DM group and control group (vertical *p*-values), and no significance was found for all comparisons. Moreover, unpaired *t*-test analysis for different genotypes among people with diabetes and the control group for all lipid profile parameters was performed (horizontal *p*-values), significance was detected for most comparisons and comparisons with no significant differences might be due to low sample size (Table S4 in the Supplementary Materials).

### 3.5. Comparison of the Lipid Profile and FokI Polymorphism Genotypes

The results of R × C chi-square test for patients and control samples (Table 6 and Table 7, respectively) have shown no statistically significant difference in lipid profile parameters levels at different genotypes of *FokI* polymorphism (*p* > 0.05).

### 3.6. Predicting Diabetes for the Testing Set

A confusion matrix was built for the testing set for each group of the selected inputs (Table 8). Number of people with diabetes who were randomly selected in the testing set is “a + c”, and number of people with no diabetes randomly selected in the testing set is “b + d”.

Accuracy was calculated according to the following formula:

Accuracy=(TP+TN)/(TP+TN+FP+FN)=(a+d)/(a+b+c+d)

where TP is true positive; TN is true negative; FP is false positive; and FN is false negative. True positive rate (TPR or sensitivity) was calculated by the formula:

TPR=TP/(TP+FN)=a/(a+c)

while true negative rate (TNR or specificity) was calculated by the formula:

TNR=TN/(TN+FP)=d/(b+d)

In all trials, the testing set was 20% (~32 cases) of all cases (164 cases). In trail 1, an FNN was used to predict the diabetes status as output based on the *FokI* polymorphism, lipid profile, gender and age as inputs. The true positive rate was 75%, the true negative rate was 94% and the accuracy was 88% (Table 9).

Trial 2 was performed with the exclusion of age, and gender information from the input data for the FNN. The true positive rate was 77%, the true negative rate was 80% and accuracy was 79%. While in Trial 3, *FokI* polymorphism was excluded and the true positive rate was 94%, the true negative rate was 65% and accuracy was 81% (Table 9).

In Trial 4, the FNN was performed with the exclusion of *FokI* polymorphism, age and gender information (i.e., just including lipid parameters). The true positive rate was 68%, the true negative rate was 71% and accuracy was 72% (Table 9).

**Figure 2:**
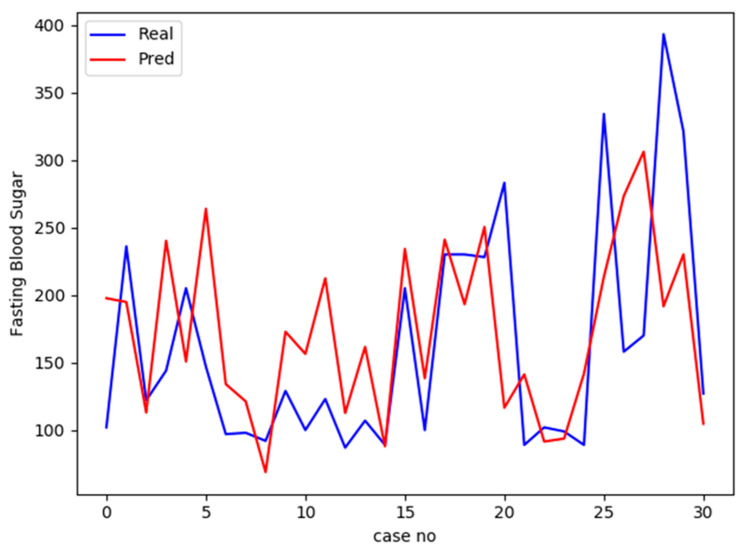
A comparison between the actual fasting blood sugar (FBS) values for and the predicted values the testing group (31 cases) using multilayer feed-forward neural network (FNN) predicting model generated based on the training set. The blue line displays actual values while the red line displays the predicted values. The *x*-axis represents case number, while *y*-axis represents the value of FBS for each case.

Afterward, the same platform of multilayer FNN was used to predict FBS instead of diabetes status. The output data in this case are continuous data, activation function “linear” was used instead of ReLU and loss function “mean squared error” was used instead of “binary crossentropy”. The correlation coefficient (r) between predicted and real values of the testing list was 0.45, which indicates a moderate uphill (positive) relationship. Figure 2 shows a comparison between predicted and real values of FBS for the testing set.

**Figure 3:**
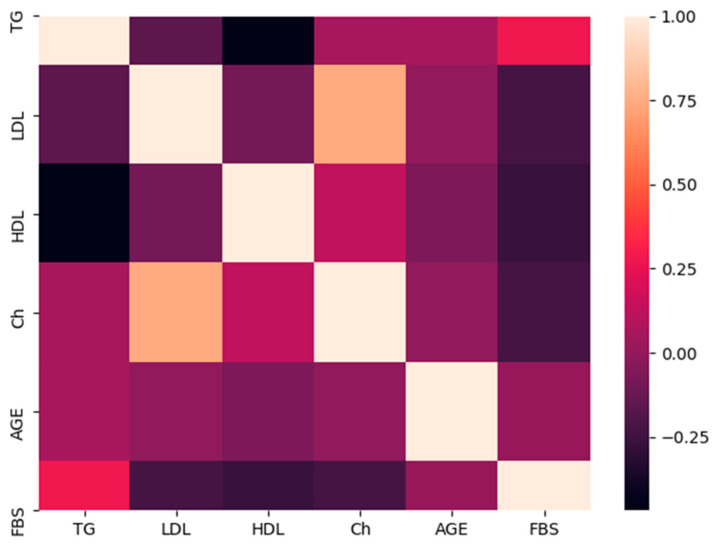
A heat map shows the associations between FBS, age and lipid parameters. Color scale is displayed at the right corner. Light colors (i.e., yellow) indicate a positive correlation, while dark colors (i.e., dark purple) indicate a negative association. FBS is moderately negatively associated with HDL, while to a lesser extent is moderately positively associated with TG.

Then, a heat map was generated for all parameters (input and output), except for those that have either binomial or discrete values (gender and *FokI* polymorphism) (Figure 3). The yellow color indicates a positive strong association and light purple shows no association, while dark purple indicates a moderate negative association. FBS is moderately negatively associated with HDL (~−0.5), while to a lesser extent is moderately positively associated with TG (~0.4).

## 4. Discussion

Vitamin D is reported to be involved in different biological processes. Variations in vitamin D endocrine regulation can lead to several common diseases, such as cardiovascular disorders, diabetes, cancer, tuberculosis and osteoarthrosis [9]. The role of vitamin D in the pathogenesis and prevention of T2DM has widespread concerns. Multiple observational studies reported a correlation between *VDR* gene polymorphisms and T2DM [2].

The purpose of this case-control study was to assess how genotypes and alleles distribution of the *VDR* gene *FokI* polymorphism affects the prevalence of T2DM in the Jordanian population. Two different major groups were considered: T2DM patients’ group and the control group. The control group did not deviate from the HWE (*p* > 0.05).

The *FokI* polymorphism, located at the 5′ end of the *VDR* gene, has been correlated with a frameshift in the *VDR* protein [35]. Therefore, the polymorphic *FokI* site in exon 2 leads to an alternative translation initiation site, which results in the addition of three amino acids to the *VDR* protein in individuals who carry the T allele [34]. Since the *FokI* polymorphism leads to a different protein, it could alter *VDR* protein function and, therefore, have some implications in T2DM susceptibility.

In the present study, various parameters were assessed in order to verify how *FokI* polymorphism influences T2DM susceptibility. Therefore, the genotype and allele distributions in patients and control groups were compared, in addition to comparing lipid profile levels. Finally, the relationship between *FokI* polymorphism genotypes and lipid profile levels in patients and control groups was analyzed.

### 4.1. Association of FokI Polymorphism Genotypes with T2DM

In this comparison, the *FokI* polymorphism and its respective genotypes were assessed. In spite of the influence of the *FokI* polymorphism on the translational activity of the *VDR* gene, which alters the structure of the *VDR* protein, there were no statistically significant results for this polymorphism (*p* > 0.05). This study demonstrated that the *FokI* polymorphism in the *VDR* gene was not associated with T2DM in the Jordanian population. The frequency of the T allele in the *FokI* polymorphism was approximately the same in T2DM patients and control subjects (27.4 and 23.8, respectively). These results fit with those shown by other studies [6,10,40,41].

The *FokI* polymorphism has been investigated in various studies on T2DM risk assessment (Table 10). Similar to our study, Bid et al. [42] demonstrated that there was no link between the *FokI* polymorphism and T2DM. Another study on Egyptian people, involving 63 patients with T2DM and 60 control samples, showed that the *FokI* variant was significantly associated with risk of T2DM only in patients with metabolic syndrome [10]. Malecki et al. [40] showed no significant difference in allele and genotype frequencies of the *FokI* polymorphism between 308 T2DM patients and 239 healthy individuals from Poland. Moreover, in a study on Tunisian population, Mahjoubi et al. [6] showed no significant correlation of the *FokI* polymorphism in the *VDR* gene and T2DM. These inconsistencies in the results between the studies may be explained by different reasons including the variation in allelic frequencies that observed in different ethnic groups. For example, the frequency of C allele was lower in Africans when compared to Caucasians and Asians [36], which partly supports our findings. Another reason is selection criteria adopted for patients and controls in different studies, in particular age, ethnicity, extent of disease, differences in the lifestyles (e.g., smoking, diet and physical activity) and the gene–gene/gene–environment interactions [6].

### 4.2. Association of Lipid Profile with T2DM

The differences in TG and HDL levels of T2DM and control samples showed significant difference (*p* < 0.05) as shown in Table 5. The levels of TG were higher in patient samples than control samples. These results are consistent with the result of Mahjoubi et al. [6], where TG level was higher in the T2DM patients compared to control group (*p* < 0.05). Furthermore, abnormal lipid metabolism and the abnormalities in blood lipids (dyslipidemia) is commonly associated with the development of T2DM.

### 4.3. Association of FokI Polymorphism Genotypes with Lipid Profile

The results showed no association between the *FokI* polymorphism genotypes and lipid profile parameters in T2DM patients and controls (*p* > 0.05). This result is consistent with the result of Bid et al. [41], where the *FokI* polymorphism was not associated with any of the lipid profile parameters. Another study by Al-Daghri et al. [2] also showed no association between lipid profile parameters and the *FokI* polymorphism.

However, this result was inconsistent with the result of Abdeltif et al. [9], where the genotype CC of the *FokI* polymorphism was significantly associated with increased levels of TC, LDL, HDL and TG in patients with gestational DM (GDM) compared to controls, and this can be attributed to ethnic and race variations and might indicate other contributing factors.

### 4.4. Prediction of Diabetes Status Using FNN

DM is a chronic pervasive condition that is data rich and with a variety of potential outcomes. Thus, diabetes is fertile ground for incorporating artificial intelligent (AI) [43,44,45,46,47]. The neural network is a math model which imitates the animal’s neural network behaviors. This model depends on the complexity of the system to achieve the purpose of processing information by adjusting the relationship between the internal nodes [48].

The artificial neural network (i.e., multilayer FNN) provides an appropriate platform for modeling the complex input–output relationship [32,33,37,38,39] between clinical (lipid profile), genetic (gene polymorphisms) and demographic (age and gender) factors and diabetes. Application of multilayer FNN to predict diabetes produced satisfactory results. The obtained results upon varying the input parameters yielded good accuracy with the inclusion of lipid parameters only (72%). More enhanced accuracy was yielded by including also the *FokI* polymorphism (79%) and all other parameters such as gender and age (88%). A moderate positive relationship between real FBS and predicted FBS values based on all parameters (*FokI* polymorphism, lipids, gender and age) was determined. FBS is affected by many environmental (i.e., lifestyle and exercises) and genetic factors; hence more factors are required in data analysis in the future to obtain more accurate predictions.

To compare our results with other studies, in one study, and for evaluation of the proposed model, the authors used the “Pima Indian Diabetes” dataset. The dataset includes the medical history of 768 patients. It considers nine different symptoms as parameters for occurrence of diabetes. Their accuracy of 85.09% proved the efficacy of the proposed work [44], and this result is comparable with our findings.

In another study by Zou et al. [48], 14 physical examination indexes: age, pulse rate, breathe, left systolic pressure (LSP), right systolic pressure (RSP), left diastolic pressure (LDP), right diastolic pressure (RDP), height, weight, physique index, fasting glucose, waistline, LDL and HDL were used to predict diabetes based on patients’ data obtained from two databases. The accuracy of prediction was as indicated in the Supplementary Materials (Table S5) based on including all the features or blood glucose only [48]. The accuracies of prediction (i.e., FNN) are close and comparable to those obtained in the present study.

**Figure 4:**
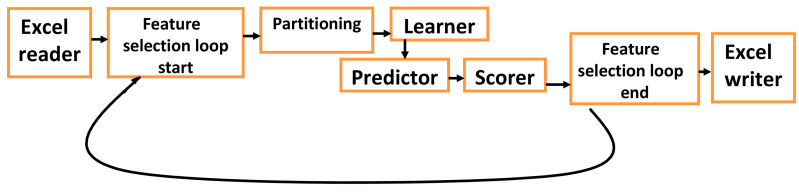
Suggested an efficient platform for medical databases with big data (large number of inputs (features)), which include clinical, demographic, socioeconomic and genetic factors. The platform starts with feeding a data set imported from the corresponding medical database. Genetic algorithm loop randomly selects different subsets (chromosomes) of feature to use them in the prediction process, it starts with “feature selection start” and ends with “feature selection end”, and this algorithm saves time in analyzing large databases. Partitioning divides data into training and testing sets, and the scorer evaluates the prediction accuracy for the testing set, the results for different chromosomes (subsets of features) can be exported and saved as output file (i.e., excel sheet). Different learners and predictors can be selected and evaluated for their performance in prediction. This platform can be embedded in the database for automatic analysis or called independently.

Neural network tools (i.e., FNN and probabilistic neural network) and machine learning tools (i.e., random forest, xgboost and decision tree) can be used with medical databases which contains clinical, demographic and genetic data [49]. For databases with large number of inputs (features), genetic algorithms can be used to randomly select subsets (chromosomes) of features (i.e., 5 to 10) which can be evaluated for their ability to predict the disease (output), and score these subsets according to their prediction accuracy (Figure 4). Genetic algorithms may shorten the time of analyzing large databases and select parameters that give the best prediction scores [50].

## 5. Limitations of the Study

The present study had some limitations. First, a larger sample size of patients and controls may be needed for further understanding the *FokI* polymorphism’s effect on T2DM. Our results were limited by the absence of both dietary information and plasma vitamin D concentrations for study participants. Studies have shown that the association between *VDR* polymorphisms and disease can vary by either past sun exposure or vitamin D level [51]. Third, polymorphisms of other *VDR* genotypes (i.e., *TaqI*, *ApaI* and *BsmI*) and their possible interactions with *FokI* variants were not evaluated.

Moreover, insulin and homeostasis model assessment-insulin resistance (HOMA-IR) were not included in the study due to the limited budget provided for this study, these parameters could be considered in the future studies. Despite of the importance of these measurements, these parameters are affected by many variables, and their inclusion in case-control studies needs to be validated and controlled for all limitations [52]. Furthermore, glycated hemoglobin (HbA1c) was measured for patients, but due to the many missing values, it was not included in the analysis. The aim of measuring HbA1c was to evaluate the association of its level with lipid profile, and we think this can be performed in future specific well-controlled studies.

The FNN training method has some limitations associated with overfitting, local optimum problems and slow convergence rate. These can be solved by cross validation (k-fold method) and using particle swarm optimization (PSO) as an evolutionary algorithm, or the AFSA-PSO-parallel-hybrid evolutionary (APPHE) algorithm to train the FNN [53]. These limitations of FNN on medical database could be investigated in the future studies, thus trade-off between speed and accuracy could be evaluated in these studies.

## 6. Conclusions

This study indicated for first time that the *FokI* polymorphism of the *VDR* gene was not associated with T2DM in the Jordanian population. Moreover, lipid profile parameters are not associated with any of the *FokI* polymorphism genotypes. It is possible that the effect of the *FokI* polymorphism on T2DM risk is specific to some ethnic populations through interaction with other environmental and clinical factors. Moreover, multilayer FNN was introduced as a modeling platform to predict diabetes status and FBS based on clinical, genetic and demographic parameters. This neural network platform can be implemented in medical databases and extended in the future for other clinical and genetic factors, which could provide an assist for early diagnosis of diabetes. Our data highlights the great value of incorporating neural network tools into large medical databases and the ability to predict patient susceptibility to different diseases.

## Figures and Tables

**Table 1:** Demographic and clinical characteristics of the study population.

Parameter	Category	T2DM Patients (*n* = 82)	Healthy Controls (*n* = 82)	χ^2^	*p*-Value
**Age (years)**	20–60	67	67	-	-
	>60	15	15	-	-
**Gender**	Male	37	37	-	-
	Female	45	45	-	-
**Lipid profile (mg/dL)**					
**HDL**	<40	46	21	13.75	0.0002 *
	≥60	6	18		
**LDL**	≤130	55	48	0.25	0.61
	>160	5	6		
**TC**	≤200	58	39	1.43	0.23
	>240	6	8		
**TG**	≤150	24	52	24.06	<0.00001 *
	>200	40	13		

HDL, high-density lipoprotein; LDL, low-density lipoprotein; TC, total cholesterol; TG, triglycerides. In the categories of each lipid profile parameter, the upper value represents the normal range, and the lower value represents the abnormal range. Chi-square (χ^2^) test was used. * Statistically significant (*p*-value < 0.05).

**Table 2:** Frequencies of different *FokI* polymorphism genotypes for healthy controls and Type 2 diabetes mellitus (T2DM) patients.

Genotype	Healthy Controls (*n* = 82)	T2DM Patients (*n* = 82)
**Mutated homozygous (CC)**	49	44
**Heterozygous (CT)**	27	31
**Wild homozygous (TT)**	6	7

**Table 3:** Hardy–Weinberg equilibrium (HWE) results for the *FokI* polymorphism and its respective genotypes in the control group.

Genotype	Observed Value	Expected Value	χ2	*p*-Value
**CC**	49	47.36	0.69	0.4
**TT**	6	4.72		
**CT**	27	29.91		

Chi-square (χ^2^) test was used. *p*-value is significant at ≤0.05.

**Table 4:** Analysis of patients (*n* = 82) vs. controls (*n* = 82) *FokI* polymorphism genotypes and alleles frequencies for T2DM patients and healthy controls.

*FokI* Polymorphism	T2DM Patients *n* (%)	Healthy Controls *n* (%)	Odds Ratio (OR)	95% CI	*p*-Value
**Genotypes**					
**CC**	44 (53.7)	49 (59.8)	CC vs. (CT and TT) 0.78	0.42–1.45	0.4
**CT**	31 (37.8)	27 (32.9)	CT vs. (CC and TT) 1.24	0.65–2.35	0.5
**TT**	7 (8.5)	6 (7.3)	TT vs. (CC and CT) 1.18	0.38–3.68	0.7
**Alleles**					
**C**	119 (72.6)	125 (76.2)	0.82	0.50–1.36	0.4
**T**	45 (27.4)	39 (23.8)	1.21	0.74–1.99	

A logistic regression model was used. *p*-value is significant at ≤0.05.

**Table 5:** Lipid profile analysis in T2DM patients (*n* = 82) and healthy controls (*n* = 82).

Parameter (mg/dL)	T2DM Patients		Healthy Controls		*p*-Value
	*D*-Value	Mean ± SD (mg/dL)	*D*-Value	Mean ± SD (mg/dL)	
**TC**	0.06 (0.91)	173.9 ± 47.2	0.08 (0.64)	201.4 ± 37.7	<0.001 *
**TG**	0.08 (0.58)	214.6 ±90.4	0.13 (0.09)	141.3 ± 77.7	<0.001 *
**HDL**	0.13 (0.13)	39.4 ± 19.7	0.07 (0.74)	51.0 ± 14.0	<0.001 *
**LDL**	0.11 (0.25)	87.8 ± 50.1	0.07 (0.73)	121.3 ± 34.6	<0.001 *

HDL, high-density lipoprotein; LDL, low-density lipoprotein; TC, total cholesterol; TG, triglycerides. * Statistically significant (*p* ≤ 0.05).

**Table 6:** Relationship between *FokI* polymorphism genotypes and lipid profile parameters in T2DM patients (*n* = 82).

Parameter (mg/dL)	Category (n)	CC	CT	TT	χ^2^	*p*-Value
**TC**	≤200 (58)	31	22	5	0.73	0.6
	>240 (6)	3	3	0		
**TG**	≤150 (23)	14	7	2	1.82	0.4
	>200 (40)	22	17	1		
**HDL**	<40 (46)	23	19	4	1.47	0.4
	≥60 (6)	4	1	1		
**LDL**	≤130 (55)	32	20	3	1.21	0.5
	>160 (5)	2	3	0		

HDL, high-density lipoprotein; LDL, low-density lipoprotein; TC, total cholesterol; TG, triglycerides. In the categories of each lipid parameter, the upper value represents the normal range, and the lower value represents the abnormal range. *p*-value is significant at ≤0.05.

**Table 7:** Relationship between *FokI* polymorphism genotypes and lipid profile parameters in controls (*n* = 82).

Parameter (mg/dL)	Category (*n*)	CC	CT	TT	χ^2^	*p*-Value
**TC**	≤200 (39)	26	9	4	1.38	0.5
	>240 (8)	5	3	0		
**TG**	≤150 (53)	34	13	6	3.35	0.1
	>200 (13)	7	6	0		
**HDL**	≥60 (18)	10	6	2	2.81	0.2
	<40 (22)	12	10	0		
**LDL**	≤130 (48)	33	11	4	2.29	0.3
	>160 (6)	3	3	0		

In the categories of each lipid parameter, the upper value represents the normal range, and the lower value represents the abnormal range. The *p*-value is significant at ≤ 0.05.

**Table 8:** General shape of the confusion matrix.

	Diabetes	No Diabetes
**Predicted diabetes**	a	b
**Predicted no diabetes**	c	d
	a + c	b + d

“a” represents the true predicted patients with diabetes, “b” represents the false predicted people with diabetes, “c” represents the false predicted people with no diabetes, and “d” represents the true predicted people with no diabetes. “a + c” represents the total number of people with diabetes, while “b+d” represents the total number of people with no diabetes.

**Table 9:** Confusion matrix for the testing set based on different combinations of input parameters.

Prediction	Trial 1		Trial 2		Trial 3		Trial 4	
	D	ND	D	ND	D	ND	D	ND
**D**	13	17	15	13	13	1	15	6
**ND**	4	3	1	6	6	12	1	11

D, diabetes; ND, no diabetes.

**Table 10:** List of studies on the *FokI* polymorphism and T2DM in different populations.

Population	Number of Controls	Number of T2DM Patients	OR (95% CI)	*p*-Value	Results	Reference
**Kashmir valley**	100	100	0.32 (0.1704–0.6154)	<0.001	Yes	[42]
**Morocco**	177	176	0.35 (0.14–0.83)	0.01	Yes	[9]
**Santiago,** **Metropolitan Region, Chile**	160	160	3.52 (1.53–8.09)	0.003	Yes	[8]
**Tunisia**	302	439	1.19 (0.63–2.25)	0.5	No	[6]
**Iraq**	75	200	0.21(0.10–0.40)	<0.0001	Yes	[4]
**Poland**	239	308	1.08 (0.85–1.37)	>0.05	No	[40]
**North India**	160	100	0.72 (0.49–1.06)	0.098	No	[41]
**Saudi**	285	285	0.73 (0.56–0.95)	0.02	Yes	[2]
**Egypt**	60	130	0.51 (0.37–0.72)	0.001	Yes	[10]
**Caribbean**	76	201	0.52 (0.30–0.90)	0.02	Yes	[11]

Yes/No indicates if the study has reported an association between this *FokI* polymorphism and T2DM or not.

## Supplementary Materials

The following are available online at https://www.mdpi.com/2079-7737/9/8/222/s1, Table S1: Participants’ data, Table S2: Association of *FokI* polymorphism with response diabetes (*n* = 164, adjusted by TG + LDL + HDL + CH + Gender + age) (performed by snpStats at www.snpstats.net/analyzer.php), Table S3: *FokI* polymorphism and gender cross-classification interaction (*n* = 164, adjusted by TG + LDL + HDL + CH + Gender + age) (performed by snpStats at www.snpstats.net/analyzer.php), Table S4: ANOVA analysis for different lipid parameters among different genotypes for people with diabetes and people with no diabetes (horizontal *p*-values), and unpaired *t*-test analysis for different genotypes among people with diabetes and people with no diabetes for all lipid profile parameters (vertical *p*-values)., Table S5: Predicting diabetes by using all feature or blood glucose.
