# Supplementary material for: Artificial Neural Networks Model for Predicting Type 2 Diabetes Mellitus Based on VDR Gene FokI Polymorphism, Lipid Profile and Demographic Data
Source: Biology (Basel). 2020 Aug 13;9(8):222. doi: 10.3390/biology9080222 (PMC7465516; doi:10.3390/biology9080222)
Supplement: Supplementary file 1 [file biology-09-00222-s001.pdf]

biology

MDPI

Table S1. Participants' data.

Diabetes Status	TG	LDL	HDL	CH	FBS	Gender	Age	*FokI* genotype
1	266	66.8	30	150	228	F	46	CC
1	273	55.4	49	159	155	M	64	CT
1	213	105.4	32	180	201	M	65	CC
1	166	179.8	43	256	257	F	51	CT
1	104	239.2	45	305	233	M	58	CT
1	253	72.4	49	172	89	F	54	CC
1	197	53.6	25	117	147	M	60	CT
1	73	72.4	53	140	249	F	55	CT
1	250	29	41	120	393	F	60	CC
1	77	143.6	49	208	84	M	50	CC
1	140	57	52	137	24	F	60	TT
1	63	48.4	31	92	310	F	56	CT
1	118	42	43	109	242	M	65	CT
1	237	28	78	154	170	M	65	CC
1	133	39	128	190	249	M	38	CC
1	70	53	37	104	401	M	39	CC
1	96	47	105	171	203	M	72	CC
1	155	28	99	158	226	M	50	TT
1	140	23	86	137	126	M	63	CT
1	142	151.6	29	209	198	M	75	TT
1	202	73.6	27	141	370	F	42	CC
1	115	163	45	231	170	F	60	CC
1	138	150.4	56	234	410	F	33	CC
1	230	178	22	246	278	M	65	CT
1	306	127	18	169	309	M	52	TT
1	160	118	51	201	122	M	80	CC
1	213	134.4	29	206	321	F	54	CT
1	306	59	16	213	144	M	60	CT
1	133	149.4	28	204	268	F	51	CC
1	200	127	31	198	334	F	47	TT
1	248	121.4	36	207	405	F	54	CC
1	294	40.2	41	140	270	F	50	CC
1	294	39.2	16	114	195	M	66	CC
1	142	60.6	25	114	230	M	57	CC
1	250	23	26	99	197	F	50	CC
1	276	133	18	196	110	M	72	CT
1	290	59	26	143	270	F	59	CC
1	484	127	23	160	201	M	52	CC
1	108	49.4	26	97	158	F	52	CC
1	338	145	26	111	230	M	37	CT
1	200	133	23	196	114	F	48	CT
1	372	81.6	29	185	219	F	49	CC
1	266	73.8	27	154	390	F	57	CC
1	296	150.8	24	234	158	F	45	CC
1	234	131.2	38	216	283	M	43	CT
1	142	198.6	37	264	129	F	44	CC
1	230	145	21	230	274	F	50	CC
1	272	117.6	33	205	351	F	46	CT
1	186	45.8	22	105	170	F	52	CT
1	187	100.4	36	187	141	F	54	CT
1	178	65.2	24.2	125	348	M	66	CC
1	295	94.1	24.9	178	174	F	50	CT
1	181	66.6	24.2	177	194	M	57	CC

1	230	6	55	107	165	F	59	CT
1	331	1.37	28.2	232	123	M	52	CT
1	226	61.7	43.9	151	136	F	52	CC
1	411	1.6	28.2	112	122	M	37	CC
1	142	83	58	195	225	F	50	CC
1	77	94.8	49.8	160	139	M	72	CC
1	174	73.6	42.6	151	205	F	48	CC
1	142	133	28	191	225	M	55	CT
1	266	145	41	109	182	F	51	CC
1	355	5	34	110	236	M	50	CC
1	186	49.4	31	193	162	F	71	TT
1	366	36.8	24	134	209	M	42	CT
1	271	116.8	30	201	119	M	58	CT
1	310	104	28	194	147	F	55	CT
1	168	83.4	50	167	273	F	54	CT
1	355	83	33	187	262	M	57	CT
1	168	135.4	58	227	135	F	48	CC
1	133	61.4	34	122	433	M	42	CC
1	90	116	31	165	355	F	54	CT
1	228	12.04	41	207	205	F	20	CC
1	81	116	50	162	138	F	41	CT
1	320	61.8	69	272	181	F	60	CC
1	265	44.8	54	191	275	M	53	CT
1	333	83	40	278	276	F	45	CC
1	391	51	41	177	475	F	55	CT
1	187	58.6	35	131	155	M	50	CC
1	170	94	47	175	201	F	53	CC
1	178	151.4	20	207	329	F	55	TT
1	111	122	56	200	127	M	57	CC
2	76	86	38	139	102	M	61	CT
2	90	93	51	162	103	M	58	CC
2	119	96	49	166	89	M	72	CC
2	557	152	32	295	132	M	71	CC
2	175	148	48	231	96	M	66	CT
2	109	108	51	181	93	F	47	CC
2	170	82	60	176	96	F	67	CC
2	125	93	76	250	92	F	36	CC
2	217	160	36	239	102	F	42	CT
2	113	143	56	222	94	F	63	CT
2	112	147	34	203	89	M	68	CT
2	102	104	58	182	95	F	47	CC
2	208	138	37	217	87	M	62	CC
2	226	102	33	184	98	M	50	CC
2	129	125	45	196	99	M	42	CC
2	135	153	36	216	101	M	51	CC
2	155	108	37	176	95	M	53	CC
2	72	113	75	202	92	F	37	CC
2	194	144	51	234	95	F	43	CC
2	119	125	54	203	92	M	49	CC
2	53	110	56	177	102	F	26	CC
2	138	158	37	223	93	M	51	CT
2	37	82	66	155	N/A	F	30	CC
2	89	121	54	193	87	M	36	TT
2	81	100	54	170	80	M	20	TT
2	209	142	32	216	103	M	42	CC
2	89	98	53	168	102	M	58	CC
2	123	120	37	182	106	M	44	CT
2	104	88	74	183	N/A	F	56	CT

2	109	106	39	167	104	M	29	CC
2	117	124	55	202	N/A	F	46	CC
2	128	110	54	190	99	M	54	TT
2	155	63	74	168	N/A	F	57	CC
2	71	75	34	123	83	M	33	CC
2	140	101	46	175	91	F	57	CC
2	91	112	80	206	92	F	31	CT
2	192	84	43	165	111	M	44	CT
2	105	130	46	197	115	M	53	CC
2	112	89	60	171	93	F	87	TT
2	103	137	45	203	100	M	61	TT
2	121	138	52	214	86	M	55	CC
2	59	143	68	227	N/A	F	40	TT
2	63	188	86	287	88	M	54	CT
2	178	110	58	204	100	M	47	CC
2	221	63	33	140	100	M	44	CT
2	133	157	59	243	99	M	54	CT
2	168	116	41	191	102	F	35	CC
2	169	116	41	191	102	F	55	CC
2	91	132	52	202	102	F	60	CT
2	248	185	25	260	95	M	35	CC
2	163	144	31	208	82	M	47	CT
2	178	97	57	190	94	F	46	CT
2	218	97	47	188	90	F	37	CC
2	86	144	65	216	91	F	50	CT
2	96	147	54	220	N/A	F	31	CC
2	106	74	53	147	92	F	77	CC
2	376	207	31	313	98	M	54	CT
2	142	132	63	227	110	F	54	CC
2	177	105	40	181	N/A	F	63	CT
2	103	104	63	188	102	F	41	CC
2	134	85	34	151	92	M	42	CC
2	74	79	47	141	97	F	57	CC
2	282	139	30	225	123	M	45	CC
2	197	82	47	168	N/A	F	34	CC
2	239	240	44	309	107	F	62	CT
2	71	194	57	270	91	M	47	CC
2	90	185	59	262	N/A	F	46	CC
2	69	132	63	209	94	F	37	CC
2	43	76	92	177	80	F	24	CT
2	78	156	42	214	116	M	42	CT
2	138	120	58	204	92	F	49	CC
2	299	110	31	201	85	M	36	CT
2	146	115	67	211	102	F	69	CC
2	208	141	53	235	N/A	F	58	CT
2	184	141	47	225	101	F	55	CT
2	142	151	47	226	129	F	70	CC
2	144	142	36	207	115	F	68	CC
2	102	80	67	195	N/A	F	41	CC
2	41	44	73	125	94	F	52	CC
2	48	112	58	180	N/A	F	38	CT
2	117	144	61	228	89	F	59	CT
2	197	113	52	204	N/A	F	49	CC

Diabetes status: 1, diabetic; 2, healthy. Results calculated in mg/dL.

Table S2. Association of *FoKI* polymorphism with response diabetes (n = 164, adjusted by TG + LDL + HDL + CH + Gender + age) (performed by snpStats at www.snpstats.net/analyzer.php).

Model	Genotype	Diabetes = 0 *n* (%)	Diabetes = 1 *n* (%)	OR (95% CI)	*P* -Value	AIC	BIC
**Codominant**	C/C	49 (59.8%)	44 (53.7%)	1.00	0.54	182	209.9
	C/T	27 (32.9%)	31 (37.8%)	1.38 (0.60–3.17)			
	T/T	6 (7.3%)	7 (8.5%)	1.98 (0.48–8.21)			
**Dominant**	C/C	49 (59.8%)	44 (53.7%)	1.00	0.32	180.2	205
	C/T-T/T	33 (40.2%)	38 (46.3%)	1.48 (0.68–3.24)			
**Recessive**	C/C-C/T	76 (92.7%)	75 (91.5%)	1.00	0.42	180.6	205.4
	T/T	6 (7.3%)	7 (8.5%)	1.75 (0.44–7.01)			
**Overdominant**	C/C-T/T	55 (67.1%)	51 (62.2%)	1.00	0.57	180.9	205.7
	C/T	27 (32.9%)	31 (37.8%)	1.26 (0.56–2.83)			
**Log-additive**	---	---	---	1.40 (0.77–2.54)	0.27	180	204.8

Table S3. FoKI polymorphism and Gender cross-classification interaction table (n = 164, adjusted by TG + LDL + HDL + CH + age) (performed by snpStats at www.snpstats.net/analyzer.php).

	**Female**			**Male**		
	**Diabetes = 0**	**Diabetes = 1**	**OR (95% CI)**	**Diabetes = 0**	**Diabetes = 1**	**OR (95% CI)**
**C/C**	29	26	1.00	20	18	0.70 (0.25–1.97)
**C/T**	14	15	1.56 (0.51–4.77)	13	16	0.83 (0.26–2.67)
**T/T**	2	4	2.06 (0.23–18.85)	4	3	1.32 (0.21–8.08)

Interaction p-value: 0.95.

Table S4. ANOVA analysis for different lipid parameters among different genotypes for people with diabetes and people with no diabetes (horizontal p-values), and unpaired t-test analysis for different genotypes among people with diabetes and people with no diabetes for all lipid profile parameters (vertical p-values).

Parameter (mg/dL)	Diabetes Status	CC	CT	TT	F-Ratio Value	*P*-Value
**TC**	**People with diabetes**	170.5 (48.9)	176.8 (79.2)	181.6 (27.4)	0.26	0.77
	**People with no diabetes**	196.2 (35.6)	212.7 (42.5)	192.3 (21.3)	1.87	0.16
	***p*-value**	0.00	0.00	0.45		
**TG**	**People with diabetes**	214.1 (93.8)	221.6 (92.6)	186.7 (57.3)	0.42	0.66
	**People with no diabetes**	142.3 (80.9)	149.8 (77.9)	95.3 (24.4)	1.22	0.30
	***p*-value**	0.00	0.00	0.00		
**HDL**	**People with diabetes**	41.8 (21.3)	36.0 (14.6)	40 (28.3)	0.80	0.45
	**People with no diabetes**	50.6 (12.8)	50.6 (17.2)	55.8 (7.7)	0.38	0.68
	***p*-value**	0.02	0.00	0.21		
**LDL**	**People with diabetes**	82.6 (47.0)	92.8 (54.4)	98.8 (52.2)	0.56	0.57
	**People with no diabetes**	115.2 (31.2)	133.4 (40.1)	116.7 (21.0)	2.57	0.08
	***p*-value**	0.00	0.00	0.45		

Table S5. Predict the diabetes by using all feature or blood glucose.

Database	Platform	Accuracy [a]	Accuracy [b]
**Luzho**	Random Forest	0.81	0.76
	J48	0.79	0.76
	FNN	0.78	0.76
**Pima Indian**	Random Forest	0.76	0.67
	J48	0.73	0.69
	FNN	0.77	0.72

[a] accuracy when all parameters are included. [b] accuracy when blood glucose just included.

© 2020 by the author. Licensee MDPI, Basel, Switzerland. This article is an open access article distributed under the terms and conditions of the Creative Commons Attribution (CC BY) license (http://creativecommons.org/licenses/by/4.0/).
